# Comparing maternal outcomes in spontaneous singleton pregnancies versus *in vitro* fertilization conception: Single-center 10-year cohort study

**DOI:** 10.5935/1518-0557.20220002

**Published:** 2022

**Authors:** Neeta Singh, Neena Malhotra, Reeta Mahey, Monika Saini, Garima Patel, Ankita Sethi

**Affiliations:** 1 Division of Reproductive Medicine, Department of Obstetrics and Gynecology, All India Institute of Medical Sciences, New Delhi, India

**Keywords:** ART, IVF, maternal outcomes

## Abstract

**Objective:**

A successful assisted reproductive technique (ART) cycle is not flawless, and several studies have reported high incidences of maternal complications, but the association is inconclusive. In addition, the racial and ethnic effects of the Asian population undergoing ART on maternal outcomes is not well studied. This study attempts to compare various maternal outcome parameters ART and spontaneously conceived singleton pregnancies from a single high volume tertiary care centre.

**Methods:**

A retrospective cohort study from a single tertiary infertility center was conducted from January 2011 to September 2020. The study included 1125 IVF conceived singletons (AP group) and 7193 spontaneous conceived singletons (SP group). The groups were compared using the Pearson Chi-square test and the adjusted odds ratio calculated using multivariate analysis.

**Results:**

Maternal outcomes like gestational hypertension, pre-eclampsia, gestational diabetes (GDM), oligohydramnios, chorioamnionitis, operative, and instrumental delivery were significantly different in the two groups (*p*<0.05). The AP group had a significantly increased risk of GDM (aOR 1.093; 95% CI 1.076-1.110) and pregnancy-induced hypertension (PIH) (aOR 1.577; 95% CI 1.288-1.930) as compared to the SP group. IVF significantly increases the risk of abruption by 2 times (*p*=0.028), and independently increases the risk of caesarean section by 3.1-fold (*p*<0.001). But overall the IVF is the protective factor for oligohydramnios (*p*=0.024).

**Conclusions:**

ART increases the likelihood of pregnancy-related maternal complications, such as PIH, GDM, abruption, chorioamnionitis, and an increased rate of caesarean delivery. Thus, all patients undergoing ART procedures should receive pre-conceptional counselling regarding the associated obstetric risks and consider ART pregnancy as a high-risk pregnancy.

## INTRODUCTION

Spontaneous conception achieves successful results in as many as 60% of couples within three cycles (about three months) and approximately 80% get pregnant with six cycles (about six months) of trying conception (Taylor, 2003). But one in six couples encounter problems with fertility, and most require some medical assistance for achieving parenthood. Since the invention of assisted reproductive technology in the late 1900s, the field has expanded exponentially with high success rates. Proving to be a boon for infertile couples, it includes complex umpteen procedures customized according to age, infertility factor, and various other prognostic variables. Although ART is a boon for infertile couples, each ART cycle is exhaustive and requires utmost vigilance as the stakes are high in terms of failure to conceive.

A successful ART cycle is not flawless and is associated with several maternal complications. This brings concern regarding the overall safety of these procedures. Various studies have reported an increased risk of gestational diabetes mellitus, gestational hypertension, preeclampsia, intrahepatic cholestasis of pregnancy, placenta previa, placental abruption, preterm premature rupture of membranes, placental adherence, postpartum hemorrhage, polyhydramnios, preterm labor, low birth weight, and small-for-date infant in pregnancies conceived after ART as compared with spontaneous conceptions (Zhu *et al*., 2016).

However, the currently available data regarding the association between maternal complications in IVF pregnancies is inconclusive and requires further exploration. The lack of a national ART registry further adds to the scarcity of data comparing ART cycles with spontaneously conceived pregnancies in a racially and ethnically distinguished Asian population. This study aimed to compare various maternal outcome parameters in IVF and spontaneously conceived singleton pregnancies over a decade from a single high volume tertiary care center.

## MATERIALS AND METHODS

A retrospective study was conducted at an IVF facility of a tertiary care centre. It was an observational cohort study involving prospectively collected data from January 2011 to September 2020, comprising all infertile females undergoing successful fresh transfer IVF cycles and resulting in a singleton pregnancy. For comparison and correlation, a similar cohort of spontaneously conceived singleton pregnancies, delivered in our center during the same period was included as a control. Inclusion criteria included either primigravida or multigravida with a past history of only one abortion, while patients with co-existing medical disorders (cardiac disease, pregestational diabetes, hypertension, asthma, seizure disorder, hypothyroidism), *in-vitro* fertilization with donor oocyte cycle, or frozen embryo transfer were excluded from the study. We had 10,360 females who fulfilled these criteria; however, due to incomplete medical records only 8,318 were included in the study. Among these, 7,193 conceived spontaneously, while the remaining 1,125 conceived via IVF. All patients received standard antenatal care as per the departmental protocol and delivered at least one live fetus after the viable period of gestation (POG) i.e., 26 weeks. Both the ART and control group were analyzed for maternal complications.

Evaluation of maternal characteristics comprised age, parity, any pregnancy induced morbidities like gestational hypertension, preeclampsia, eclampsia, gestational diabetes, polyhydramnios, oligohydramnios, chorioamnionitis, and antepartum hemorrhage due to placenta previa or abruption.

### Statistical analysis

The data was compiled on an Excel spreadsheet and analyzed using the SPSS.v.23 software. The cohort was divided into two groups: (a) AP Group: females conceived via ART and (b) SP Group: spontaneously conceived females. Variables amongst the group were compared using the Pearson Chi-square test and a *p*-value <0.05 was considered statistically significant. Multivariate analysis was performed; adjusted odds ratios (aORs), 95% confidence intervals (CIs), and 2-sided *p-*values were calculated. Differences were considered statistically significant if the effect estimate excluded 1.0 from the 95% CI and the 2-sided *p*-value was <0.05.

## RESULTS

A total of 8,318 females were included in the study of which 7,193 females conceived spontaneously, while 1,125 required ART. The mean age of the SP group was 26±4.42 years; whereas it was 31±5.24 years in the AP group. The mean age, as well as the distribution among various subgroups, was significantly different between the two groups, as shown in Table 1 (*p*<0.001). Most of the patients (>50%) in ART group were more than 30 years of age.

**Table 1:** Maternal age distribution in the two groups.

Maternal age	AP Group (ART conception) N=1125 (%)	SP Group (Spontaneous conception) N=7193 (%)	*p*-value
18-20 years	5 (0.4%)	308 (4.3%)	<0.001
21-25 years	109 (9.7%)	2827 (39.3%)	<0.001
26-30 years	391 (34.8%)	3029 (42.1%)	<0.001
31-35 years	388 (34.5%)	841 (11.7%)	<0.001
36-40 years	168 (14.9%)	167 (2.3%)	<0.001
>40 years	64 (5.7%)	21 (0.3%)	<0.001

### Pregnancy induced complications

While the incidence of gestational hypertension, pre-eclampsia, gestational diabetes, chorioamnionitis, rate of caesarean delivery, and abruptio placentae was significantly higher (*p*<0.05), the rate of oligohydramnios was significantly lower in the AP group (*p*=0.034), as shown in Table 2. However, the risk of polyhydramnios, eclampsia, and placenta previa did not differ between the two groups (*p*>0.05).

**Table 2:** Pregnancy induced complications in IVF singletons and spontaneously conceived controls population.

Complications	AP Group (ART conception) N=1125	SP Group (Spontaneous conception) N=7193	*p*-value
Gestational hypertension	156 (13.9%)	506 (7.0%)	<0.001
Preeclampsia (PE)	37 (3.3%)	120 (1.7%)	<0.001
Eclampsia	2 (0.2%)	26 (0.4%)	0.323
Gestational diabetes	217 (19.3%)	663 (9.2%)	<0.001
Oligohydramnios	23 (2.0%)	231 (3.2%)	0.034
Polyhydramnios	5 (0.4%)	40 (0.6%)	0.635
Abruptio placentae	12 (1.1%)	40 (0.6%)	0.043
Placenta previa	6 (0.5%)	47 (0.7%)	0.638
Mode of delivery Normal delivery Caesarean section Forceps/vacuum delivery	228 (20.3%) 873 (77.6%) 24 (2.1%)	3501 (48.7%) 3083 (42.9%) 609 (8.5%)	<0.001
Chorioamnionitis	32 (2.8%)	3 (0.01%)	<0.001

### IVF as an independent risk factor for Gestational Diabetes Mellitus (GDM)

Various contributory factors, such as type 2 diabetes mellitus (DM) and previous history of GDM were eliminated using the exclusion criteria. The unadjusted OR (Odds ratio) for GDM in pregnancies after IVF conception was 2.35, but after adjusting for known confounder (Age) the ART increases the risk of GDM by 1.09 times (*p*<0.001) (Table 3).

**Table 3:** Multivariate analysis of risk factors for GDM in the present study.

Multivariate analysis		
Variable	OR (95%CI range)	*p*-value
Maternal age	1.495 (1.239-1.804)	<0.001
Mode of conception (IVF vs. SC)	1.093 (1.076-1.110)	<0.001

### IVF as an independent risk factor for Pregnancy-induced hypertension (PIH)

PIH is defined as systolic blood pressure (SBP) >140 mmHg and diastolic blood pressure (DBP) >90 mmHg. It includes both gestational hypertension and pre-eclampsia. There are various risk factors of PIH, such as chronic hypertension, renal disease, type 2 DM, and multiple pregnancies but pregnancies with these risk factors were excluded from the study. Unadjusted OR for PIH in pregnancies after IVF conception was 2.04. But after adjusting for the confounding factor (Age), IVF increases the risk of PIH by 1.5 times, as shown in Table 4.

**Table 4:** Multivariate analysis of risk factors for PIH in the present study.

Multivariate analysis		
Variable	OR (95%CI Range)	*p*-value
Maternal age	1.051 (1.034-1.069)	<0.001
Mode of conception (IVF vs. SC)	1.577 (1.288-1.930)	<0.001

### IVF as an independent risk factor for Abruptio Placentae

The AP group had a higher incidence of Abruptio Placentae as compared to the SP group (1.1% *vs*. 0.6%). On adjusting for various co-variates like pre-eclampsia and gestational hypertension, IVF significantly increases the risk of abruption by 2-fold (*p*=0.028) (Table 5).

**Table 5:** Multivariate analysis of risk factors for Abruptio Placentae in the present study.

Multivariate analysis		
Variable	OR (95%CI Range)	*p*-value
Pre-eclampsia	1.232 (0.164-9.270)	0.839
Gestational hypertension	0.407 (0.096-1.720)	0.222
Mode of conception (IVF vs. SC)	2.070 (1.080-3.968)	0.028

### IVF as an independent risk factor for Polyhydramnios

The incidence of polyhydramnios is similar between the two comparative groups (0.4% *vs*. 0.6%; *p*=0.635). Even after adjusting for the confounding factors like gastrointestinal malformations, GDM, and neural tube defects, IVF does not increase the risk of polyhydramnios (*p*=0.560) (Table 6).

**Table 6:** Multivariate analysis of risk factors for polyhydramnios in the present study.

Multivariate analysis		
Variable	OR (95%CI Range)	*p*-value
Gastrointestinal malformations	30.834 (12.355-76.951)	<0.001
GDM	1.681 (0.738-3.825)	0.216
Neural tube defects	23.567 (5.283-105.125)	<0.001
Mode of conception (IVF vs. SC)	0.755 (0.294-1.938)	0.560

### IVF as an independent risk factor for Oligohydramnios

The incidence of oligohydramnios was significantly lower in the AP group as compared to the SP group (2% *vs*. 3.2%; *p*=0.034). On multivariate analysis, IVF seems to be a protective factor for oligohydramnios in the AP group (*p*=0.024) (Table 7).

**Table 7:** Multivariate analysis of risk factors for oligohydramnios in the present study.

Multivariate analysis		
Variable	OR (95%CI Range)	*p*-value
Pre-eclampsia	2.244 (1.147-4.387)	0.018
Gestational hypertension	1.139 (0.728-1.783)	0.570
Genito-urinary malformations	11.615 (5.878-22.954)	<0.001
Mode of conception (IVF vs. SC)	0.605 (0.391-0.936)	0.024

### IVF as an independent risk factor for caesarean section

The current trend of increasing the number of C-sections is well-depicted in the AP group, where 77.6% of the deliveries were by C-section. APH, placenta previa, pre-eclampsia, oligohydramnios, maternal age, and IVF are independent risk factors for caesarean section as depicted on Table 8.

**Table 8:** Multivariate analysis of risk factors for caesarean section in the present study.

Multivariate analysis		
Variable	OR (95%CI Range)	*p*-value
Antepartum Hemorrhage	7.461 (2.868-19.412)	<0.001
Placenta previa	14.587 (4.462-47.685)	<0.001
Pre-eclampsia	3.012 (2.062-4.401)	<0.001
Oligohydramnios	3.012 (2.062-4.401)	<0.001
Maternal age	1.093 (1.081-1.105)	<0.001
Mode of conception (IVF *vs*. SC)	3.158 (2.701-3.692)	<0.001

## DISCUSSION

With the advent of the 21^st^ century and significant advances in culture media and IVF procedures, ART has proven to be a boon for infertile couples. Reported success rates can be as high as 35-40%, but it does not come without adverse effects. This retrospective cohort study attempted to analyze the overlooked aspects of ART adverse antenatal outcomes in fresh transfer IVF/ICSI cycles of conceived pregnancies from a single tertiary care center in the South-east Asian region.

With emerging globalization and gender equality, a trend of delayed conception until the late 3^rd^ to early 4^th^ decade of life is becoming pervasive (Mills *et al*., 2011). Also evident in this study, the average age of IVF-conceived females is 31 years and more, so >50% are above 30 years of age. The average age of spontaneously conceived females being 26 years emphasizes that increasing age predisposes to a heightened need for ART. Advanced maternal age further predisposes to increased risk of prematurity, preeclampsia, abruption, placenta previa, and adverse perinatal outcomes (Blomberg *et al*., 2014).

The existing knowledge pool supports an association between PIH and IVF conception, however conclusive evidence remains contentious regarding causation between the two. A metanalysis (Almasi-Hashiani *et al*., 2019) including 156,246 ART cases, of which 14,560 developed preeclampsia (PE) reported a significant correlation between ART and risk of PE, the same is also reflected in the current study. Several metanalyses have also reported an increased risk of PIH/PE irrespective of the type of IVF cycle or singleton/multiple pregnancies (Palomba *et al*., 2016; Pandey *et al.*, 2012). While Xiong *et al*. (2017)reported no differential risk concerning the development of PE/PIH between fresh or thawed IVF cycle, Luke *et al*. demonstrated a 1.3 fold increased risk of PE with thawed IVF cycles and no association with fresh cycles (Luke *et al*., 2019). Contrary to the aforementioned studies, the fresh transfer IVF cycle significantly predisposed patients to PIH/PE in our study. This conflicting result may stem from several independent risk factors associated with PIH, including age >35 years, primigravida, nulliparity, previous history of abortion, twin pregnancy, or pre-existing hypertension/diabetes mellitus (Hinkosa *et al.*, 2020). As most of these variables were either excluded or adjusted in the current study, we conclude that the fresh transfer IVF cycle acts as an independent risk factor for the occurrence of PIH/PE.

Multiple studies have propounded an increased risk of gestational diabetes in ART pregnancies (Grady *et al*., 2012; Zhu *et al*., 2016). Various risk factors interplay in the pathogenesis of GDM, which comprises positive family history, high parity, advanced maternal age, multiple pregnancy, and hypothyroidism. In the current study, after ruling out the aforementioned risk factors, ART was found to independently increase the risk of GDM by 1.09-fold; however, the exact mechanism remains elusive.

ART involves frequent manipulation of gametes, thereby potentially disrupting the epigenetic reprogramming of the embryo, thus affecting both embryonic and extraembryonic tissues (Vrooman *et al.*, 2016). Consequently, edema and microcalcifications also occur in the placenta. Transmission electron microscopic examination of the ART placenta has demonstrated degenerative changes in terminal villi, decreased apical microvilli, and increased multiple vacuoles (Zhang *et al*., 2011) These changes result in placenta-mediated complications, reportedly: antepartum hemorrhage (APH), abruptio placenta, and placenta previa. In tandem with our study, a metanalysis by Pandey *et al.* (2012), analyzing 20,807 IVF conceptions reported an increased risk of APH in IVF pregnancies. There is a fourfold increased risk of placental abruption and a twofold increased risk of placenta previa reported in ART conceived pregnancies (Zhu *et al.*, 2016) resulting from unwarranted excessive release of prostaglandins after mechanical stimulation during embryo transfer, leading to implantation in the lower uterine segment (Baba *et al*., 2000). As an institute protocol, there is strict adherence to standard guidelines to transfer embryo 1-1.5 cm below the uterine fundus (Toth *et al*., 2017), thus limiting the incidence of placenta previa to 0.5% in our study population. The above-mentioned uteroplacental insufficiency/placental vascular abnormalities also lead to amniotic fluid disorders (Schucker *et al*., 1996; Beall *et al*., 2007). Oligohydramnios being the most common association with either singletons or twin pregnancies (Katalinic *et al*., 2004). However, Zhu *et al*. (2016) reported otherwise, with decreased risk of oligohydramnios in singleton IVF pregnancies, as noted in our study too. But our study reported no association between polyhydramnios and IVF conception, while an increased risk for the same was documented by Zhu *et al*. (2016). A possible explanation for this phenomenon remains strict glycemic control in patients with gestational diabetes mellitus as a part of protocol-based antenatal care, thus preventing excess amniotic fluid formation in IVF-conceived females.

IVF babies are usually deemed “precious” and therefore there is a high maternal request for a safer delivery option mainly a caesarean section (Minkoff & Berkowitz, 2005). This is also agreed upon by 40 to 54% of gynecologists due to fear associated with adverse outcomes in a high-risk precious pregnancies (Bergholt *et al*., 2004; Rivo *et al*., 2018). 77.6% of IVF-conceived pregnancies in our study were delivered via cesarean section. This adds to the economic burden for the couples over and above the expensive assisted reproduction techniques (Gillet *et al*., 2011).

Suspected chorioamnionitis was clinically diagnosed in 2.8% of IVF pregnancies, with the speculated mechanism of intra-uterine contamination being introduced during the IVF procedure. Some case reports have also described similar findings with increased risk of chorioamnionitis leading to preterm birth; however, precise explanation for this rare occurrence requires studies with a larger cohort (Ganer Herman *et al.*, 2015).

This study evaluated outcomes of IVF conceptions from a high volume tertiary care center in a country that has the second largest population and houses more than a million IVF facilities providing infertility services at a much lower cost compared to the western world (Bansode, 2017). This comparative series describes the largest cohort studied amongst any of the developing nations. Although studies with a much larger cohort have been published, due to the lack of an established national registry only this sample size could be achieved. This study highlighted various associations between IVF and maternal complications in an ethically and racially unique Asian population. This would provide a benchmark to create awareness among fertility specialists, especially from developing nations regarding various possible complications related to IVF and would wary them to be more vigilant in diagnosing as well as treating these patients.

## CONCLUSION

With the rising incidence of infertility, assisted reproductive techniques are gaining popularity with a steep rise in ART conceptions. Although ART has been a boon for many distressed couples, the procedure is not without complications. Through this study, an attempt to create awareness and sensitization among reproductive specialists and high-risk pregnancy clinicians is made to optimise the patients before recruitment in IVF because few ill-fated ART births are associated with poorer maternal outcomes. According to evidence, ART increases the likelihood of pregnancy-related maternal complications comprising PIH, GDM, abruption, chorioamnionitis, and increased rate of cesarean delivery. Thus, all patients undergoing ART procedures should receive pre-conceptional counselling regarding the associated obstetric risks and consider ART-pregnancy as a high-risk pregnancy. Also, caregivers are advised to maintain a close vigil during the antepartum and immediate postpartum period to balance the risk of complications and successful conception.
